# Pickering Emulsion Stabilized by Hordein–Whey Protein Isolate Complex: Delivery System of Quercetin

**DOI:** 10.3390/foods13050665

**Published:** 2024-02-22

**Authors:** Songqi Yang, Yunan Jin, Feifan Li, Jinfeng Shi, Jiahui Liang, Xiaohong Mei

**Affiliations:** 1 College of Food Science & Nutritional Engineering, China Agricultural University, Beijing 100083, China; songzisq@163.com (S.Y.); jinyn923@163.com (Y.J.); sjf970421@163.com (J.S.); ljhshixiannv@163.com (J.L.); 2 Key Laboratory of Safety Assessment of Genetically Modified Organism (Food Safety), Ministry of Agriculture and Rural Affairs of the People’s Republic of China, Beijing 100083, China; 3 College of Food Science and Engineering, Shandong Agriculture and Engineering University, Jinan 250100, China; lifeifan5503@163.com; 4 Center of Food Colloids and Delivery of Functionality, College of Food Science and Nutritional Engineering, China Agricultural University, Beijing 100083, China

**Keywords:** hordein, whey protein isolate fibril, Pickering emulsion, quercetin

## Abstract

As a lipophilic flavonol, quercetin has low bioavailability, which limits its application in foods. This work aimed to prepare a hordein-based system to deliver quercetin. We constructed hordein–whey isolate protein fibril (WPIF) complexes (H-Ws) by anti-solvent precipitation method at pH 2.5. The TEM results of the complexes showed that spherical-like hordein particles were wrapped in WPIF clusters to form an interconnected network structure. FTIR spectra revealed that hydrogen bonds and hydrophobic interactions were the main driving forces for the complex formation. H-W_1_ (the mass ratio of hordein to WPIF was 1:1) with a three-phase contact angle of 70.2° was chosen to stabilize Pickering emulsions with oil volume fractions (φ) of 40–70%. CLSM images confirmed that the oil droplets were gradually embedded in the three-dimensional network structure of H-W_1_ with the increase in oil volume fraction. The emulsion with φ = 70% showed a tight gel structure. Furthermore, this emulsion exhibited high encapsulation efficiency (97.8%) and a loading capacity of 0.2%, demonstrating the potential to deliver hydrophobic bioactive substances. Compared with free quercetin, the bioaccessibility of the encapsulated quercetin (35%) was significantly improved. This study effectively promoted the application of hordein-based delivery systems in the food industry.

## 1. Introduction

Quercetin is a lipophilic flavonol which widely exists in fruits and vegetables [1]. Due to its diverse beneficial bioactivities (such as antioxidant, anti-hypertensive, and anti-diabetic), quercetin has received increasing attention in recent years [2]. However, the low bioavailability and the poor chemical stability severely limit its applications in the food and pharmaceutical industries [3]. Several delivery systems have been developed to increase the chemical stability and bioavailability of quercetin, including Pickering emulsions [4], liposomes [5], hydrogels [6,7], nanoparticles [8], cyclodextrin complexations [9]. In comparison with traditional emulsions, food-grade colloid particle (e.g., proteins, polysaccharides, lipids) stabilized Pickering emulsions exhibit promising prospects in encapsulating hydrophobic bioactive substances due to good biocompatibility and high loading capacity [10].

Hordein, the main storage protein in barley seeds, contains a high proportion of hydrophobic amino acids [11]. This prolamin can self-assemble into nanoparticles in aqueous solution, presenting the potential to be exploited as Pickering emulsion stabilizers. However, the strong hydrophobicity of a single hordein makes it difficult to prepare stable oil-in-water (O/W) Pickering emulsions [12]. Previous results illustrated that the hydrophobicity of hordein was notably tuned by combining it with hydrophilic polysaccharide: chitosan and the formed composite particles were used to fabricate stable Pickering emulsions [13]. So far, the research on complexes between hordein and other hydrophilic macromolecules as Pickering emulsion stabilizers has not been reported.

Whey protein isolate (WPI), the main byproduct of dairy industry, tends to self-assemble into an amyloid-like fibrillar structure by the acid-heat method [14]. Compared with the native protein, the viscosity, gel property and emulsifying ability of whey protein isolate fibril (WPIF) are remarkably improved [15,16]. And WPIF has been applied as a stabilizer of Pickering emulsions to deliver bioactive substances [17]. Therefore, it is worth exploring the feasibility of constructing stable hordein/WPIF complex-stabilized O/W Pickering emulsion.

In this study, based on the simple and economical advantages of the anti-solvent precipitation method [18], it was used to construct hordein/WPIF complexes. The physical properties (microstructure, surface hydrophobicity. etc.) of the complexes were characterized. At the same time, interface and rheological properties of hordein/WPIF complex stabilized Pickering emulsions were also investigated. The ability of the emulsions to deliver quercetin was further evaluated. This work will greatly promote the application of hordein in food industries.

## 2. Materials and Methods

### 2.1. Materials

Hulled barley and soybean oil were purchased from the local market. WPI (protein content: 93.0%) was provided by Hilmar Cheese Company, Inc. (Merced, CA, USA). Quercetin (purity: 97%) was purchased from Shanghai Yuanye Biotechnology Co., Ltd. (Shanghai, China). Pepsin was obtained from VWR Life Sciences Co., Ltd. (Radnor, PI, USA). Pancreatin, lipase, and bile salt were purchased from Sigma-Aldrich (St. Louis, MI, USA).

### 2.2. Extraction of Hordein

**Figure 1:**
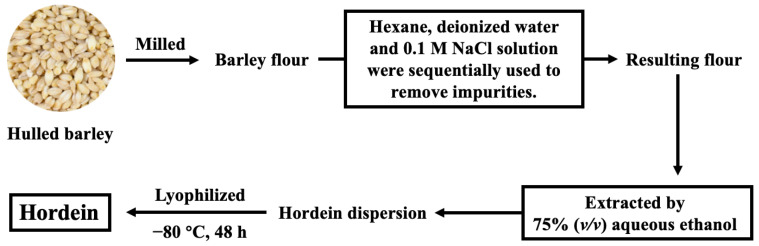
The process of hordein extraction.

The extraction of hordein was performed according to our previous study [13]. As presented in Figure 1, hulled barley was milled into flour, and the impurities of the resulting flour were removed sequentially by hexane, deionized water and 0.1 M NaCl solution. Hordein was then extracted by 75% (*v*/*v*) aqueous ethanol. The resulting hordein dispersion was lyophilized for 48 h and stored at 4 °C for future analysis.

### 2.3. Preparation of WPIF

WPIF was prepared as described by Cui et al. [19]. Ten grams of WPI was dissolved in 400 mL of deionized water and stirred for 12 h to ensure protein hydration. Then, the WPI solution was acidified to pH 2.0 using 5 M HCl. After being heated at 80 °C for 22 h, the resulting WPIF solution (2.5 wt%) was immediately cooled in an ice bath and subsequently stored at 4 °C for further use.

### 2.4. Preparation of Hordein–WPIF Complexes

Hordein–WPIF complexes (H-Ws) were prepared using the anti-solvent precipitation method. A hordein solution (2.5 wt%) was obtained by dissolving hordein in 75% (*v*/*v*) aqueous ethanol solution, and the pH of the solution was adjusted to pH 2.5. Under mild stirring, the hordein solution was dropped into the WPIF solution with the mass ratios of 1:1, 1:2, 1:3, 1:4, and 1:5. After removing the ethanol by rotary evaporation, the protein content of each sample was balanced to 2.5 wt%. The obtained complexes were labeled as H-W_1_, H-W_2_, H-W_3_, H-W_4_, and H-W_5_, respectively.

### 2.5. Zeta-Potential Measurement of H-Ws

After being diluted to 0.1 mg/mL, the zeta potential of each dispersion sample was determined using a Nano ZS Zetasizer (Malvern Instruments Ltd., Worcestershire, UK). The measurement was performed at room temperature.

### 2.6. Fluorescence Spectroscopy

The fluorescence intensities of hordein, WPIF, and H-Ws were determined by an F-7000 fluorescence spectrophotometer (Hitachi, Tokyo, Japan). The protein content of every dispersion sample was adjusted to 0.2 mg/mL. The emission spectra ranged from 300 to approximately 400 nm under a fixed excitation wavelength of 280 nm, with a scanning speed of 240 nm/min.

### 2.7. Surface Hydrophobicity

The surface hydrophobicity values of hordein, WPIF, and H-Ws were investigated using ANS as a fluorescence probe. The ANS solution (8.0 mM) was prepared by dissolving ANS in PBS (0.01 M, pH 7.0). The concentrations of the sample solutions were adjusted with a range from 0.01 to 0.5 mg/mL. After that, each sample solution was mixed with 20 μL of the ANS solution and kept for 2 min in the darkness. The fluorescence intensities of the resulting solutions with the different concentrations were measured at the excitation wavelength of 390 nm, and the emission wavelength was set at 470 nm. The hydrophobicity intensity (H_0_) of each sample was defined as the slope of linear fitting between fluorescence intensity and sample concentration.

### 2.8. Fourier Transform Infrared Spectroscopy (FTIR)

The infrared spectra of hordein, WPIF, and H-Ws were recorded using a Spectrum 100 Fourier transform infrared spectrometer (Perkin-Elmer, Warrington, UK). Briefly, 2.0 mg of a lyophilized sample was mixed with 200 mg dry potassium bromide; then, the mixtures were pressed into pellets. The wavenumber ranged from 4000 to 400 cm^−1^ with a resolution of 4 cm^−1^.

### 2.9. Transmission Electron Microscopy (TEM)

The microstructures of WPIF and H-Ws were observed by a transmission electron microscope (JEM-1200EX, Japan Electronics Co., Ltd., Tokyo, Japan). Briefly, 10 μL of freshly prepared sample dispersions (0.5 wt%) were dropped onto the carbon-coated copper grid. Then, the copper grid was dried at 25 °C. The voltage of TEM was 100 kV during imaging.

### 2.10. Wettability Measurement

The wettability of samples (WPIF and H-Ws) was investigated by determining their three-phase contact angles (θ) with an OCA 20 AMP contact angle measuring instrument (Dataphysics Instruments GmbH, Stuttgart, Germany). Briefly, the lyophilized samples were pressed into tablets. Each tablet was immersed into soybean oil. Then, 5 μL of deionized water was dropped on the surface of the tablet, and the drop images were obtained. The θ of the sample was computed on the basis of the Laplace–Young equation.

### 2.11. Preparation of H-W_1_ Stabilised Pickering Emulsions

Pickering emulsions with various oil volume fractions (φ = 40–70%) stabilized by H-W_1_ were prepared at a complex content of 2.5 wt%. The oil phase (soybean oil) and H-W_1_ dispersions were mixed, and the mixture system were homogenized with a homogenizer (PhD Technology LLC, Saint Paul, MN, USA) (11,000 rpm, 40 s) to obtain emulsions.

### 2.12. Droplet Size Measurement

The droplet size of Pickering emulsions (φ = 40–70%) was determined with a LS230 laser particle size analyzer (Beckman Coulter, Inc., Brea, CA, USA). The parameters of refractive indices were 1.33 and 1.52 for water and oil, respectively. The average volume-weighted diameter (D_4,3_) was utilized to characterize droplet sizes. All the tests were performed at room temperature.

### 2.13. Microstructure Measurement

The microstructures of emulsion were monitored using a LSM 800 confocal laser scanning microscope (CLSM) (Carl Zeiss AG, Oberkochen, Germany). Nile Blue A (1 mg/mL) and Nile Red (1 mg/mL) were separately added for staining. The corresponding mixtures were reacted for 30 min in the darkness. The excitation wavelengths used for Nile Blue A and Nile Red were, separately, 633 nm and 488 nm.

### 2.14. Rheological Properties Measurements

The rheological properties of the emulsions were investigated with a Discovery HR-2 dynamic shear rheometer (TA Instruments, Newcastle, DE, USA). The varying trend of apparent viscosity was monitored as the shear rate varied from 0.1 to 100 s^−1^. Frequency sweep measurements were performed at 0.1% strain with angular frequency increasing from 0.1 to 100 rad⋅s^−1^. The change in the elastic modulus (G′) and the loss modulus (G″) was recorded.

### 2.15. Storage Stability of Pickering Emulsions

The emulsions were separately kept in sample vials for 14 days at 25 °C. The storage stability of the emulsions was investigated based on appearance, droplet size (measured by the method of Section 2.12) and creaming index (CI). CI of the corresponding emulsions after 14 days was determined based on the following equation:

(1) $$CI\left(\%\right)=\frac{H_{e}}{H_{t}}\times 100$$

where H_e_ is the height of the emulsion layer (cm) and H_t_ is the total height of the emulsion (cm).

### 2.16. Preparation of Quercetin-Loaded Pickering Emulsions

Quercetin was dispersed in soybean oil at the ratio of 19:100 (mass/volume) and stirred at 4 °C for 4 h. According to the method of Section 2.11, quercetin-loaded Pickering emulsions (70% oil volume fraction) stabilized by H-W_1_ (2.5 wt%) were prepared. Encapsulation efficiency (EE) and loading capacity (LC) of quercetin were assessed as described by the previously reported method [20,21]. Briefly, a 100 μL quercetin-loaded emulsion was diluted to 10 mL with ethanol, vortexed for 1 min and then centrifuged (10,000× *g*, 10 min). The absorbance of the collected supernatant at 374 nm was measured using a TU-1901 ultraviolet-visible spectrometer (Beijing Puxi General Instrument Co., Ltd., Beijing, China). The encapsulated quercetin content was calculated by means of calibration curve (y = 0.107x − 0.1691, R^2^ = 0.9990). EE and LC of quercetin were determined on the basis of the following equations:

(2) $$EE\left(\%\right)=\frac{W_{e}}{W_{t}}\times 100$$

(3) $$LC\left(\%\right)=\frac{W_{e}}{W_{p}}\times 100$$

where W_e_ and W_t_ represent the encapsulated quercetin content (mg) and the total quercetin content (mg), and W_p_ represents the weight of the soybean oil in the emulsion.

### 2.17. Quercetin Stability Measurements

In this study, the storage, thermal and photochemical stabilities of quercetin were evaluated, respectively. The free quercetin dissolved in soybean oil was used as control. The storage stability of quercetin was examined by maintaining the quercetin-loaded emulsion at room temperature for 15 days, and the quercetin content in the emulsions was measured using the method in Section 2.16. The retention rate of quercetin was calculated by the ratio of the quercetin content on Day t to the quercetin content on Day 0. To assess the photochemical stability of quercetin, the emulsions were exposed to UV light (40 W, 254 nm) with a vertical distance of 1 cm for 6 h. The retention rate of quercetin was determined for every 2 h. Additionally, the emulsions were heated for 6 h at 80 °C in the darkness. The thermal stability of quercetin was evaluated by calculating retention rate for every 2 h.

### 2.18. In Vitro Digestion

The in vitro digestion behaviors of quercetin-loaded emulsion were evaluated according to Shen et al. [22].

Simulated gastric fluid (SGF) digestion: The emulsion was added to SGF (containing 2.0 g/L sodium chloride solution and 3.2 g/L pepsin) at a 1:1 ratio (*v*/*v*), and the pH of the system was adjusted to 2.0. The system was incubated (100 rpm) for 2 h at 37 °C.

Simulated intestinal fluid (SIF) digestion: After SGF digestion, the pH value of the system was adjusted to 7.0 and mixed with SIF (consisting of 16.7 g/L calcium chloride solution, 99.4 g/L sodium chloride solution, 17.0 g/L bile salt, 5.5 g/L pancreatin and 5.5 g/L lipase) in a 3:1 ratio (*v*/*v*). The mixture was incubated (100 rpm) at 37 °C for 2 h.

In the course of SIF digestion, the pH value of the system was maintained at 7.0 ± 0.1 by way of adding 0.1 mol/L sodium hydroxide solution. The amount of free fatty acids (FFA) was calculated by recording the sodium hydroxide solution volume based on the following equation:

(4) $$FFA\left(\%\right)=\frac{M_{lipid}{\times C_{NaOH}\times V}_{NaOH}}{2\;W_{lipid}}\times 100$$

where M_lipid_ is the mean molecular weight of soybean oil (880 g/mol), C_NaOH_ is the molarity of sodium hydroxide (M), V_NaOH_ is the volume of sodium hydroxide solution (mL), and W_lipid_ is the mass of the oil phase (g) in the digestion system.

In addition, the bioaccessibility of quercetin during intestinal digestion was also measured. Intestinal digestion was centrifuged (15,000× *g*, 30 min), then the micellar phase was collected. Quercetin bioaccessibility was calculated via the ratio of the initial content of quercetin to the content of quercetin in the micellar phase.

### 2.19. Statistical Analysis

Each experiment was repeated at least in triplicate. Data were analyzed using SPSS 26.0 software (IBM Crop., Westchester, NY, USA), and statistical differences were evaluated by ANOVA with Duncan post-test. The data were shown as the mean ± standard deviation, which were considered to have significant differences when *p* < 0.05.

## 3. Results and Discussion

### 3.1. Zeta Potential of H-Ws

**Figure 2:**
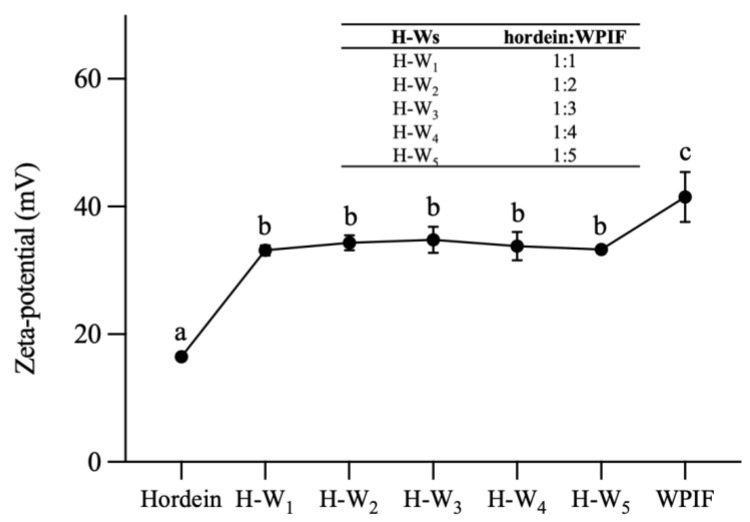
Zeta potential of hordein, WPIF and H-Ws at different hordein-to-WPIF mass ratios. Different letters demonstrate significant differences (*p* < 0.05).

Figure 2 demonstrates that the zeta potential value of hordein was 16.4 ± 0.9 mV at pH 2.5, which is attributed to the pH of the hordein dispersion (2.5) being lower than the isoelectric point of this protein (PI ≈ 6.5) [11]. The zeta potential value of WPIF was 41.5 ± 3.9 mV, which was in accordance with the result of Jiang et al. [15]. The zeta potential value of H-Ws was about 34 mV, which was notably higher than that of a single hordein. This difference may be ascribed to the coating of WPIF on the hordein surface. And this hypothesis was further confirmed by the TEM results as described in Section 3.2. At the same time, the zeta potential values of the complexes did not differ significantly (*p* > 0.05), which was in accordance with the findings of Zhan et al. [23]. Compared with the single WPIF, H-Ws showed lower potential values. This situation was probably due to the lower WPIF concentration in H-Ws compared with the single WPIF solution, resulting in the fewer positively charged groups located on the complex surface.

### 3.2. The Analysis of TEM

**Figure 3:**
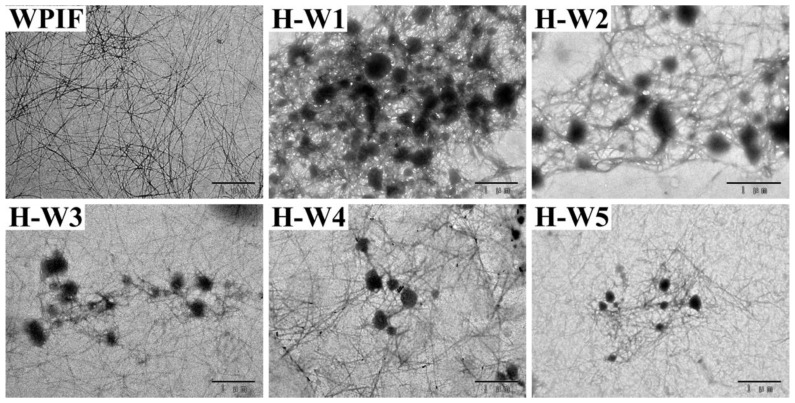
Transmission electron microscopy images of WPIF and H-Ws. Scale bar: 1 μm.

The morphological structure of H-Ws was observed by TEM, and the results are presented in Figure 3. WPIF exhibited a filamentous structure with a high length-to-diameter ratio (about 100:1–500:1), which agreed with the previous studies [15]. The images of H-Ws indicated that spherical-like hordein particles, which were produced through anti-solvent precipitation [13], were wrapped in WPIF clusters, leading to the formation of an interconnected network structure. With the increasing content of WPIF, the size of hordein particles in H-Ws gradually decreased. The increase in the fibril proportion might have strengthened steric hindrance between the complexes, further restraining hordein aggregation [24].

### 3.3. Surface Hydrophobicity of H-Ws

**Figure 4:**
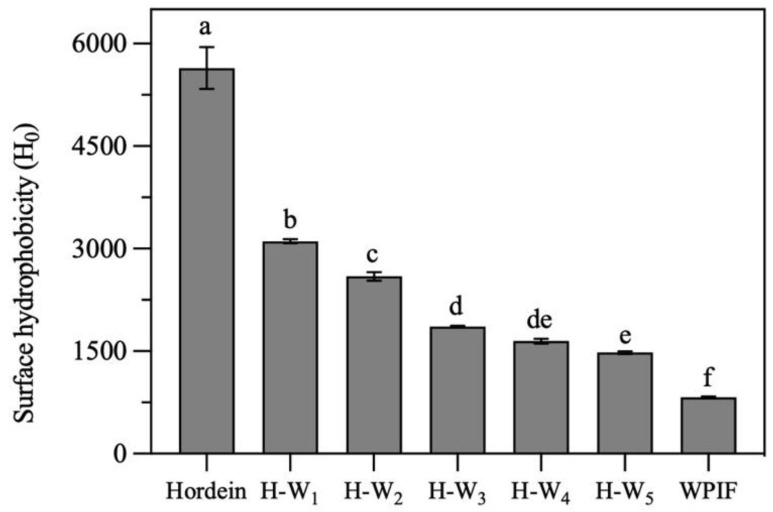
Surface hydrophobicity of hordein, WPIF and H-Ws. Different letters demonstrate significant differences (*p* < 0.05).

Surface hydrophobicity is one of the key structural parameters to investigate surface properties of proteins. As presented in Figure 4, the surface hydrophobicity of hordein was significantly higher than that of the single WPIF. In comparison with that of the single hordein, the surface hydrophobicity of H-Ws significantly decreased with the addition of WPIF. It was attributed to the fact that some hordein particles were wrapped inside hydrophilic WPIF (as shown in Section 3.2), leading to the decrease in exposure of hydrophobic groups of hordein. These results confirmed that the addition of WPIF effectively improved the hydrophily of hordein.

### 3.4. FTIR of H-Ws

**Figure 5:**
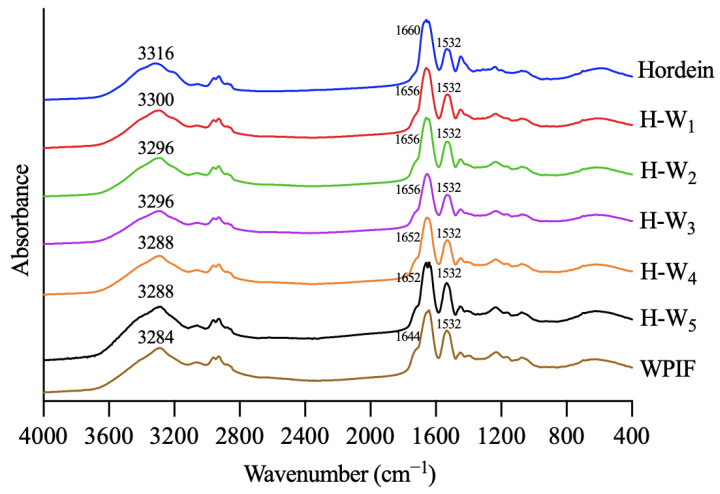
Fourier transform infrared spectra of hordein, WPIF, and H-Ws.

Infrared spectroscopy was used to investigate interactions between hordein and WPIF [25]. As shown in Figure 5, hordein and WPIF separately exhibited characteristic peaks at 3316 cm^−1^ and 3284 cm^−1^, which were ascribed to the stretching vibration of O–H [26]. Nevertheless, these characteristic peaks of H-Ws shifted to 3300 cm^−1^ (H-W_1_), 3296 cm^−1^ (H-W_2_ and H-W_3_), and 3288 cm^−1^ (H-W_4_ and H-W_5_), demonstrating that hydrogen bonds were formed between hordein and WPIF [27]. The infrared spectra of hordein showed two characteristic peaks at 1660 cm^−1^ and 1532 cm^−1^, separately corresponding to the amide I band (the stretching vibration of the C=O group) and the amide II band (the stretching vibration of the C–N group and the bending vibration of the N–H group) [28]. Characteristic peaks of WPIF corresponding to the amide I band and the amide II band were observed at 1644 cm^−1^ and 1532 cm^−1^. In comparison with single hordein and WPIF, the amide I band of H-Ws was separately shifted to 1656 cm^−1^ (H-W_1_, H-W_2_, and H-W_3_) and 1652 cm^−1^ (H-W_4_ and H-W_5_), suggesting possible existence of hydrophobic interactions between WPIF and hordein [29], which was consistent with the findings reported by Wei et al. [30].

### 3.5. Fluorescence Spectra of H-Ws

**Figure 6:**
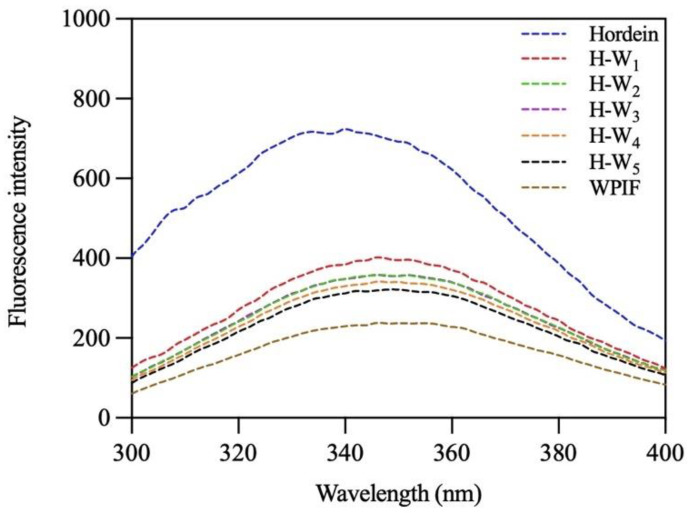
Fluorescence spectra of hordein, WPIF, and H-Ws.

The interaction between hordein and WPIF was further investigated by analysis of their intrinsic fluorescence spectra [31]. As presented in Figure 6, after excitation at 280 nm, hordein and WPIF separately exhibited maximum emission peaks at 340 nm and 346 nm. These results were ascribed to the fluorescence emission of tryptophan residues [32]. In comparison with hordein, the maximum emission wavelength of H-Ws was red-shifted to 346 nm, indicating the polarity increase in the micro-environment of tryptophan residues [33]. Furthermore, the addition of WPIF led to a gradual decrease in the fluorescence intensities of H-Ws, which might have been be due to the fact that hydrogen bonds or hydrophobic interaction between hordein and WPIF reduced the exposure of some tryptophan residues, further causing fluorescence quenching [34]. Fluorescence results agreed with the findings of FTIR.

### 3.6. Wettability of H-Ws

**Figure 7:**
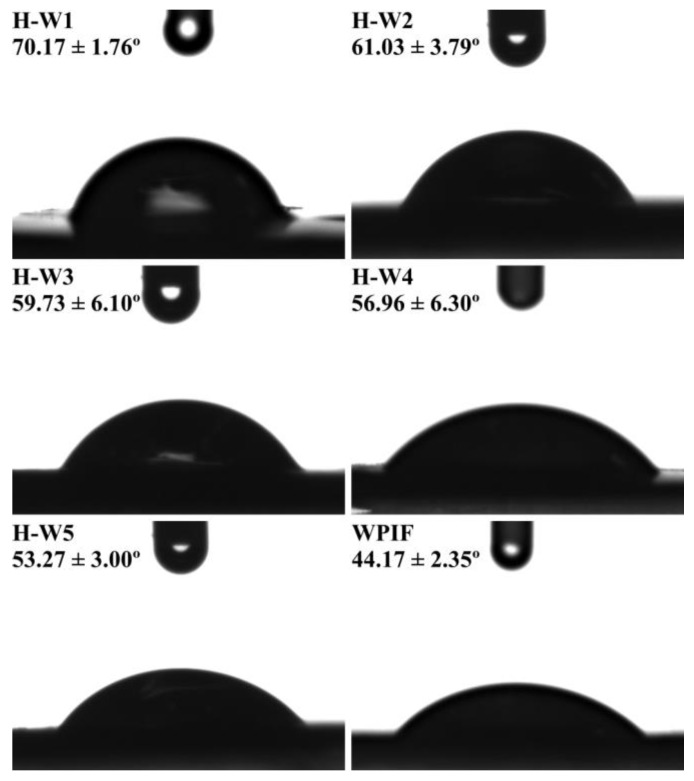
The θ of H-Ws and WPIF.

The wettability of solid particles at the oil–water interface is a key parameter to screen an ideal Pickering stabilizer [10]. Figure 7 shows that the θ of WPIF was about 44.17°, indicating its strong hydrophilicity. According to the previous study in our lab, hordein with the θ of 116.5° exhibited high hydrophobic property [13]. The θ of H-Ws decreased from 70.17° to 53.27° with the increase in the WPIF content, suggesting that the addition of WPIF improved the hydrophilicity of hordein. This may be attributed to the interactions (hydrogen bonds or hydrophobic interaction) between hordein and WPIF, which was confirmed by fluorescence and FTIR results. This situation was in agreement with the surface hydrophobicity result of H-Ws (as described in Section 3.3). In this study, due to the fact that the θ of H-W_1_ was closer to 90°, it was chosen as the following Pickering stabilizer [35].

### 3.7. Characterization of H-W_1_ Stabilized Pickering Emulsions

**Figure 8:**
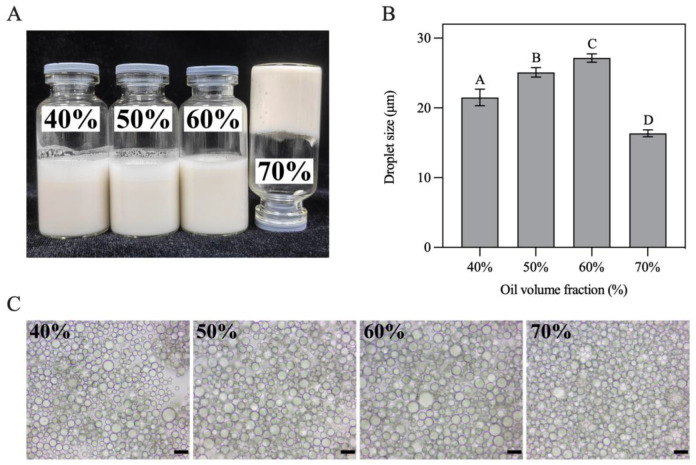
(**A**) Appearance, (**B**) droplet size and (**C**) optical microscopy images of Pickering emulsions. Different letters in (**B**) demonstrate significant differences (*p* < 0.05). In (**C**), the scale bar represents 20 μm.

In this study, H-W_1_ stabilized Pickering emulsions with various oil volume fractions (φ = 40–70%) were prepared. As presented in Figure 8A, all obtained emulsions had a homogeneous texture. The emulsion with φ = 70% presented excellent self-supporting ability when inverted, revealing the formation of a gel-like structure [36]. Figure 8B demonstrates that the size of emulsion droplets gradually increased with increasing oil volume fractions (in the φ range of 40–60%), indicating that higher oil concentration led to inadequate Pickering stabilizers absorbing on all the droplet surfaces [37]. When φ attained 70%, the size of the emulsion droplets significantly decreased. This situation might have occurred due to stronger shear forces during homogenization being needed to compensate the viscosity increase in the system, resulting in more droplets being disrupted [20]. Optical microscopy images (Figure 8C) present a similar trend of droplet size change.

### 3.8. Analysis of CLSM Images

**Figure 9:**
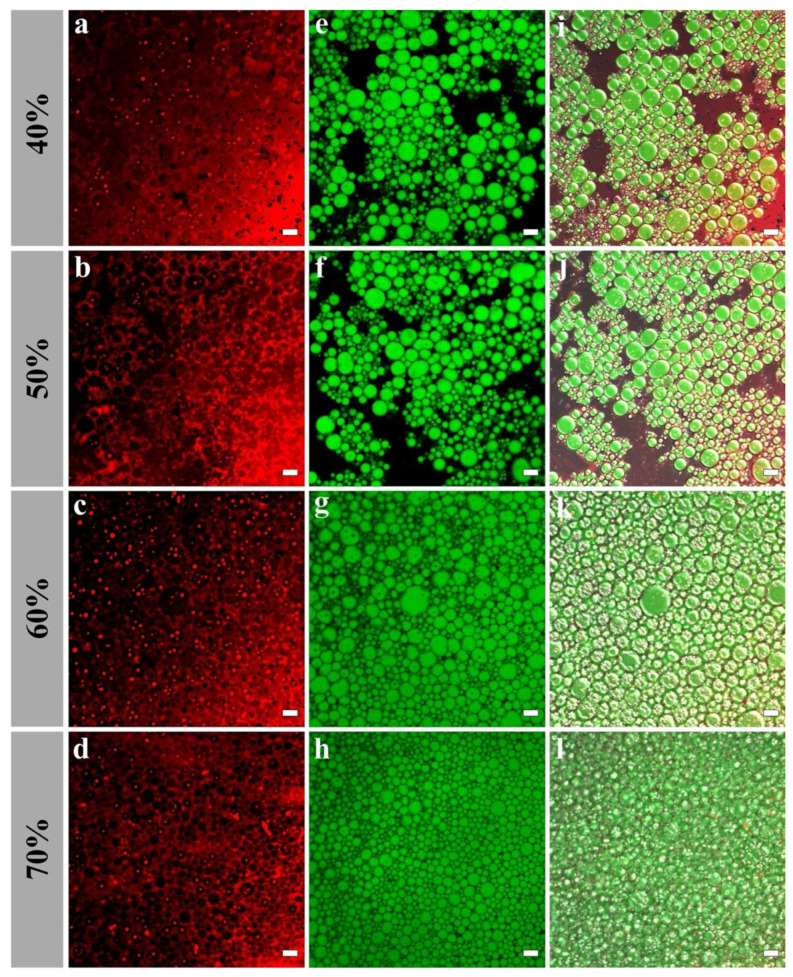
Confocal laser scanning microscope images of Pickering emulsions, (**a**–**d**) represent H-W_1_ stained by Nile Blue A, (**e**–**h**) represent oil stained by Nile Red, (**i**–**l**) represent combined images. Scale bar: 20 μm.

The interfacial microstructures of H-W_1_ (2.5 wt%) stabilized Pickering emulsions were analyzed by CLSM. As presented in Figure 9, in the emulsions with 40% and 50% oil volume fractions, the majority of H-W_1_ formed the dense network structure in the water phase. Simultaneously, the rest of H-W_1_ surrounded the oil droplets. As the oil volume fraction continued to increase, the oil droplets were gradually embedded in the three-dimensional network structure of H-W_1_, and they were tightly connected. This further led to the formation of a strong gel-like structure [38].

### 3.9. Rheological Properties

**Figure 10:**
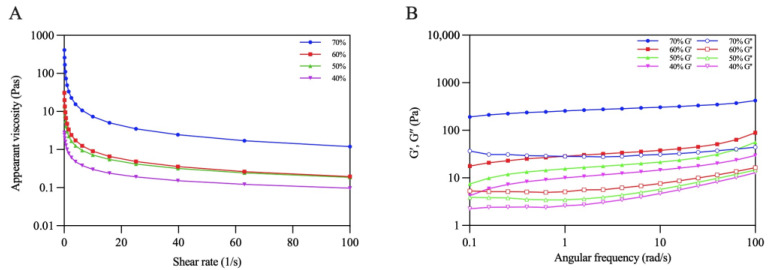
Rheological properties: (**A**) Apparent viscosity (**B**) G′ and G″ of Pickering emulsions.

The rheological behaviors of the emulsions were evaluated using steady shear flow and dynamic oscillatory tests. Figure 10A shows that all the emulsions displayed a decrease in apparent viscosity with an increasing shear rate, presenting the shear-thinning behavior of non-Newtonian fluids [39]. Moreover, the viscosity of the emulsions gradually increased with increasing oil volume fractions. The viscosity of the emulsion reached its maximum when φ increased to 70%. This phenomenon might have occurred due to the increasing emulsion stress resistance caused by the dense packing of the oil droplets under higher oil volume fraction [40].

Dynamic oscillatory tests are normally used to characterize the gel properties of Pickering emulsions. The change trends of G′ and G″ with angular frequency were recorded. As shown in Figure 10B, the G′ values of all the emulsions were higher than those of G″ when the frequency varied from 0.1 to approximately 100 rad/s, which revealed that the elastic properties were dominant in the emulsions, and the emulsions tended to form an elastic gel-like structure [37]. Additionally, both G′ and G″ increased as the oil volume fraction increased, reflecting that the gel networks of the emulsions were gradually strengthened. Similar results were found in previous research reported by Ji et al. [41].

### 3.10. Storage Stability of Pickering Emulsions

**Figure 11:**
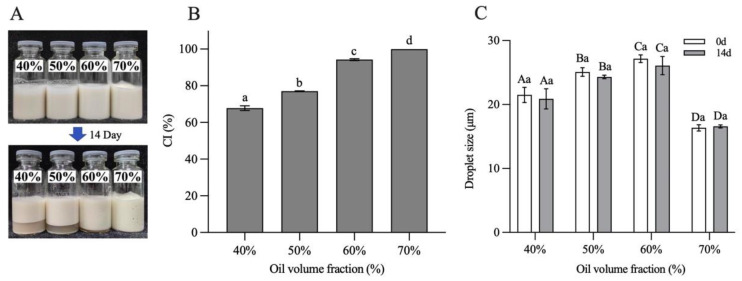
Storage stability of Pickering emulsions after 14 days. (**A**) Appearance, (**B**) creaming index and (**C**) droplet size. Different letters in (**B**) demonstrate significant differences (*p* < 0.05). In (**C**), different capital letters demonstrate significant differences between different emulsions at the same storage time, and different lowercase letters demonstrate significant differences between the same emulsion at different storage times (*p* < 0.05).

The storage stability of H-W_1_ (2.5 wt%) stabilized emulsions was assessed by investigating CI and droplet size of the emulsions after 14 days of the storage period. Figure 11 shows the results. After 14 days of storage, the emulsions with φ = 40–60% exhibited apparent phase separation (Figure 11A), and the CI values separately decreased to 58–89% (Figure 11B). However, the emulsion with φ = 70% remained homogeneous without any phase separation, corresponding to a CI value of 100%. In addition, compared with the freshly prepared emulsions, the droplet size of all emulsions did not change significantly after storage (Figure 11C), demonstrating their good storage stability [13]. The above CI and droplet size results of the emulsions indicated that the emulsion with φ = 70% exhibited better storage stability, explained by the fact that the self-supporting gel structure restricted the aggregation and floating of oil droplets. Therefore, during the following experiments, the emulsion with the oil volume fraction of 70% was chosen to encapsulate quercetin.

### 3.11. Physicochemical Stability of Quercetin

**Figure 12:**
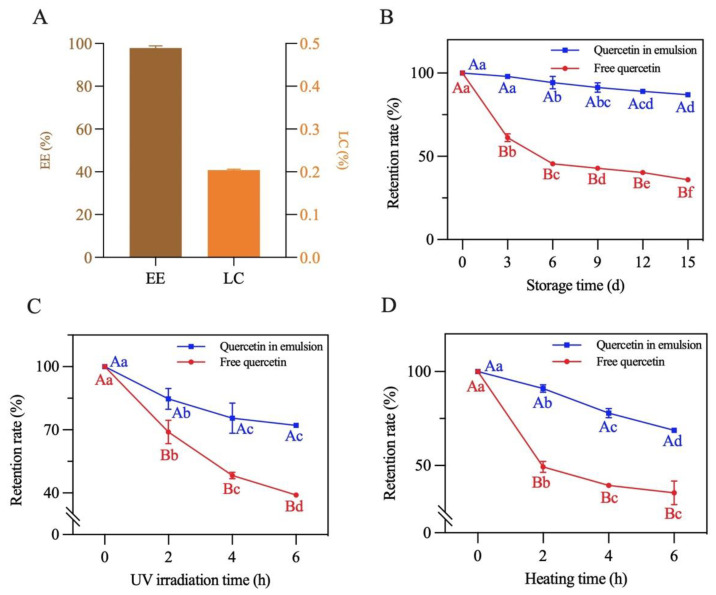
The (**A**) encapsulation efficiency and loading capacity, (**B**) storage stability, (**C**) photochemical stability and (**D**) thermal stability of quercetin loaded in the Pickering emulsion. Different lowercase letters indicate that the same carrier had significant differences at different times (*p* < 0.05), and different capital letters indicate that different carriers had significant differences at the same time (*p* < 0.05).

The EE and LC of quercetin in the emulsion with a 70% oil volume fraction were measured. As presented in Figure 12A, EE and LC concentrations were 97.8% and 0.2%, respectively, suggesting that this emulsion can be explored as a potential system for delivering bioactive components [22].

The storage, photochemical and thermal stability of quercetin encapsulated in the emulsion were measured. As shown in Figure 12B–D, the retention rate of both the loaded and free quercetin gradually decreased under the different treatment conditions. After a 15-day storage period at room temperature, 6 h of UV irradiation and 6 h of heating at 80 °C, the retention rate of the encapsulated quercetin separately decreased to 87.2%, 72.1% and 68.7%. By contrast, the retention rate of quercetin dispersed in soybean oil decreased to 35.9%, 39.0% and 35.5%, respectively. The above results reflected that physicochemical stability of quercetin loaded in the emulsion significantly improved. This was mainly due to the formation of a dense three-dimensional network structure of the complexes at the oil–water interface of the emulsion (as described in Section 3.8). This structure prevented quercetin from being directly destructed by oxygen, UV light and high temperature, effectively slowing quercetin degradation [20]. Similar results were also found in the study of Shen et al. [22].

### 3.12. Lipid Digestion and Bioaccessibility of Quercetin

**Figure 13:**
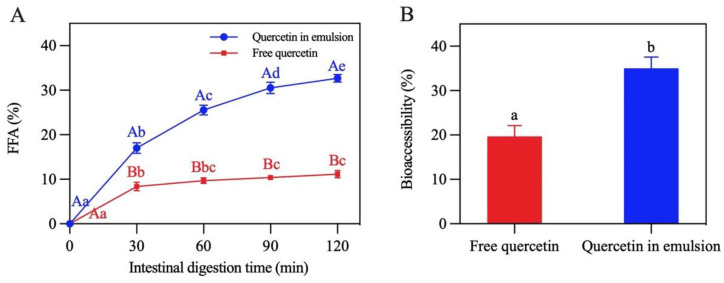
(**A**) Release of free fatty acids. (**B**) Bioaccessibility of quercetin. In (**A**), different capital letters demonstrate that different carriers have significant differences in the same digestion time (*p* < 0.05), and different lowercase letters indicate that the same carrier has significant differences in different digestion times (*p* < 0.05). The different letters in (**B**) demonstrate significant differences (*p* < 0.05).

Lipid digestion in the Pickering emulsion was studied by monitoring the release of FFA in the period of SIF digestion [42]. Figure 13A shows that the lipid digestion curve in the emulsion was steeper than that in the control group (soybean oil). When digestion reached the end point (120 min), the FFA release rate of the emulsion was 32.7%, which was almost three times that of the control group (11.1%), revealing that the formation of the emulsion remarkably accelerated lipid digestion. This was mainly because the oil phase in the Pickering emulsion was dispersed in droplets, which promoted the contact of enzymes with the oil [17].

The bioaccessibility of quercetin was characterized by determining the amount of quercetin in the micelles at the end of digestion. As presented in Figure 13B, compared with free quercetin (19.7%), the bioaccessibility of encapsulated quercetin significantly improved (35.0%). This may be due to the fact that oil droplets in this emulsion had a larger area exposed to lipase, trypsin, and bile salt, leading to the formation of more micelles for transporting quercetin to the release site [43,44]. The bioaccessibility of quercetin was found to be positively correlated with the release of FFA, which was similar to the findings reported by Yi et al. [45].

## 4. Conclusions

Hordein–WPIF complexes (H-Ws) were successfully constructed using the anti-solvent precipitation method in this study. The hydrophilicity and surface wettability of hordein were significantly improved via combination with WPIF, and hydrogen bonds and hydrophobic interaction were the main binding forces between WPIF and hordein. H-W_1_ was chosen as a stabilizer to prepare Pickering emulsions with 40–70% oil volume fractions due to its excellent wettability. Rheological and CLSM results confirmed that the emulsion with φ = 70% formed a strong gel-like structure, so this emulsion kept excellent stability after 14 days of storage. Additionally, the physicochemical stability and the bioaccessibility of quercetin encapsulated in this emulsion were notably improved. According to the above facts, this hordein-based delivery system can be explored to encapsulate other hydrophobic bioactive substances with poor physicochemical stability. In addition, this emulsion with a gel-like structure can be applied to partially hydrogenated vegetable oils and animal fats in foods. In future work, the pH, thermal, and ionic stability of this emulsion need to be evaluated to accelerate its application in real food systems. In a word, this study provided a novel way for delivering hydrophobic bioactive substances and promoted the application of hordein-based delivery systems in the food industry.

## Data Availability

The original contributions presented in the study are included in the article, further inquiries can be directed to the corresponding author.

## References

[1] Kaşıkcı M., Bağdatlıoğlu N. (2016). Bioavailability of Quercetin. Curr. Res. Nutr. Food Sci..

[2] Kandemir K., Tomas M., McClements D.J., Capanoglu E. (2022). Recent Advances on the Improvement of Quercetin Bioavailability. Trends Food Sci. Technol..

[3] Wang X., Xie H., Shi C., Dziugan P., Zhao H., Zhang B. (2021). Fabrication and Characterization of Gel Beads of Whey Isolate Protein–Pectin Complex for Loading Quercetin and Their Digestion Release. Gels.

[4] Jia X., Ma P., Taylor K.S.-Y., Tarwa K., Mao Y., Wang Q. (2023). Development of Stable Pickering Emulsions with TEMPO-Oxidized Chitin Nanocrystals for Encapsulation of Quercetin. Foods.

[5] Melchior S., Codrich M., Gorassini A., Mehn D., Ponti J., Verardo G., Tell G., Calzolai L., Calligaris S. (2023). Design and Advanced Characterization of Quercetin-Loaded Nano-Liposomes Prepared by High-Pressure Homogenization. Food Chem..

[6] Dey M., Ghosh B., Giri T.K. (2020). Enhanced Intestinal Stability and pH Sensitive Release of Quercetin in GIT through Gellan Gum Hydrogels. Colloids Surf. B.

[7] Lee T., Chang Y.H. (2020). Structural, Physicochemical, and in-Vitro Release Properties of Hydrogel Beads Produced by Oligochitosan and de-Esterified Pectin from Yuzu (*Citrus junos*) Peel as a Quercetin Delivery System for Colon Target. Food Hydrocoll..

[8] Zhang X., Wei Z., Sun Y., Luo T., Xue C. (2023). Preparation of Core–Shell Hordein/Pectin Nanoparticles as Quercetin Delivery Matrices: Physicochemical Properties and Colon-Specific Release Analyses. Food Res. Int..

[9] Wang W., Liu Y., Zhang H., Ling D., Yan Q., Wu Y., Jin Y., Xie F. (2023). Preparation of Inhalable Quercetin-β-Cyclodextrin Inclusion Complexes Using the Supercritical Antisolvent Process for the Prevention of Smoke Inhalation-Induced Acute Lung Injury. J. CO2 Util..

[10] Albert C., Beladjine M., Tsapis N., Fattal E., Agnely F., Huang N. (2019). Pickering Emulsions: Preparation Processes, Key Parameters Governing Their Properties and Potential for Pharmaceutical Applications. J. Control. Release.

[11] Li F., Huang K., Luo Y., Mei X. (2022). Isolation of B-Constituent through Selective Complex-Induced Precipitation of Hordein with ι-Carrageenan. Int. J. Biol. Macromol..

[12] Song J., Sun C., Gul K., Mata A., Fang Y. (2021). Prolamin-based Complexes: Structure Design and Food-related Applications. Compr. Rev. Food Sci. Food Saf..

[13] Li F., Li X., Huang K., Luo Y., Mei X. (2021). Preparation and Characterization of Pickering Emulsion Stabilized by Hordein-Chitosan Complex Particles. J. Food Eng..

[14] Zhao Y., Wang C., Lu W., Sun C., Zhu X., Fang Y. (2021). Evolution of Physicochemical and Antioxidant Properties of Whey Protein Isolate during Fibrillization Process. Food Chem..

[15] Jiang F., Pan Y., Peng D., Huang W., Shen W., Jin W., Huang Q. (2022). Tunable Self-Assemblies of Whey Protein Isolate Fibrils for Pickering Emulsions Structure Regulation. Food Hydrocoll..

[16] Xu X., Zhang Y., Han M., Guo Q. (2024). Whey Protein Fibrils Enhance Fat-Related Texture of Emulsion Systems: Translating Structural Changes to Textural Perception. Food Hydrocoll..

[17] Jiang F., Chen C., Wang X., Huang W., Jin W., Huang Q. (2022). Effect of Fibril Entanglement on Pickering Emulsions Stabilized by Whey Protein Fibrils for Nobiletin Delivery. Foods.

[18] Caicedo Chacon W.D., Verruck S., Monteiro A.R., Valencia G.A. (2023). The Mechanism, Biopolymers and Active Compounds for the Production of Nanoparticles by Anti-Solvent Precipitation: A Review. Food Res. Int..

[19] Cui F., McClements D.J., Liu X., Liu F., Ngai T. (2022). Development of pH-Responsive Emulsions Stabilized by Whey Protein Fibrils. Food Hydrocoll..

[20] Han S., Cui F., McClements D.J., Ma C., Wang Y., Wang X., Liu X., Liu F. (2022). Enhancing Emulsion Stability and Performance Using Dual-Fibrous Complexes: Whey Protein Fibrils and Cellulose Nanocrystals. Carbohydr. Polym..

[21] Wang N., Zhang K., Chen Y., Hu J., Jiang Y., Wang X., Ban Q. (2023). Tuning Whey Protein Isolate/Hyaluronic Acid Emulsion Gel Structure to Enhance Quercetin Bioaccessibility and In Vitro Digestive Characteristics. Food Chem..

[22] Shen S., Chen Y., Yu W., Bu Q., Fu J., Pan Z., Wang Y. (2023). High Internal Phase Pickering Emulsions Stabilized by Modified Sturgeon Myofibrillar Protein for Quercetin Delivery. Food Hydrocoll..

[23] Zhan X., Dai L., Zhang L., Gao Y. (2020). Entrapment of Curcumin in Whey Protein Isolate and Zein Composite Nanoparticles Using pH-Driven Method. Food Hydrocoll..

[24] Liu Q., Cheng J., Sun X., Guo M. (2021). Preparation, Characterization, and Antioxidant Activity of Zein Nanoparticles Stabilized by Whey Protein Nanofibrils. Int. J. Biol. Macromol..

[25] Xue J., Zhang Y., Huang G., Liu J., Slavin M., Yu L. (2018). (Lucy) Zein-Caseinate Composite Nanoparticles for Bioactive Delivery Using Curcumin as a Probe Compound. Food Hydrocoll..

[26] Cerqueira M.A., Souza B.W.S., Teixeira J.A., Vicente A.A. (2012). Effect of Glycerol and Corn Oil on Physicochemical Properties of Polysaccharide Films—A Comparative Study. Food Hydrocoll..

[27] Raeisi S., Ojagh S.M., Quek S.Y., Pourashouri P., Salaün F. (2019). Nano-Encapsulation of Fish Oil and Garlic Essential Oil by a Novel Composition of Wall Material: Persian Gum-Chitosan. LWT.

[28] Ozturk O.K., Salgado A.M., Holding D.R., Campanella O.H., Hamaker B.R. (2023). Dispersion of Zein into Pea Protein with Alkaline Agents Imparts Cohesive and Viscoelastic Properties for Plant-Based Food Analogues. Food Hydrocoll..

[29] Song J., Sun C., Xiang Y., Xie Y., Mata A., Fang Y. (2020). Fabrication of Composite Structures of Lysozyme Fibril–Zein Using Antisolvent Precipitation: Effects of Blending and pH Adjustment Sequences. J. Agric. Food Chem..

[30] Wei Y., Zhan X., Dai L., Zhang L., Mao L., Yuan F., Liu J., Gao Y. (2021). Formation Mechanism and Environmental Stability of Whey Protein Isolate-Zein Core-Shell Complex Nanoparticles Using the pH-Shifting Method. LWT.

[31] Huang S., He J., Han L., Lin H., Liu G., Zhang W. (2020). Zein-Polyglycerol Conjugates with Enhanced Water Solubility and Stabilization of High Oil Loading Emulsion. J. Agric. Food Chem..

[32] Liu H., Zhang Y., Zhang J., Xiong Y., Peng S., McClements D.J., Zou L., Liang R., Liu W. (2022). Utilization of Protein Nanoparticles to Improve the Dispersibility, Stability, and Functionality of a Natural Pigment: Norbixin. Food Hydrocoll..

[33] Shen C., Chen W., Li C., Chen X., Cui H., Lin L. (2022). Pickering Emulsion Stabilized by Gliadin/Soybean Polysaccharide Composite Colloidal Nanoparticle: Physicochemical Properties and Its Application on Washing of Fresh-Cut Cabbage. Food Res. Int..

[34] Meng R., Wu Z., Xie Q.-T., Cheng J.-S., Zhang B. (2021). Preparation and Characterization of Zein/Carboxymethyl Dextrin Nanoparticles to Encapsulate Curcumin: Physicochemical Stability, Antioxidant Activity and Controlled Release Properties. Food Chem..

[35] Chevalier Y., Bolzinger M.-A. (2013). Emulsions Stabilized with Solid Nanoparticles: Pickering Emulsions. Colloids Surf. A.

[36] Sun C., Wang C., Xiong Z., Fang Y. (2021). Properties of Binary Complexes of Whey Protein Fibril and Gum Arabic and Their Functions of Stabilizing Emulsions and Simulating Mayonnaise. Innov. Food Sci. Emerg. Technol..

[37] Li Q., Wu Y., Shabbir M., Pei Y., Liang H., Li J., Chen Y., Li Y., Li B., Luo X. (2021). Coalescence Behavior of Eco-Friendly Pickering-MIPES and HIPEs Stabilized by Using Bacterial Cellulose Nanofibrils. Food Chem..

[38] Zhao T., Huang K., Luo Y., Li Y., Cheng N., Mei X. (2023). Preparation and Characterization of High Internal Phase Pickering Emulsions Stabilized by Hordein-Chitosan Composite Nanoparticles. Colloids Surf. A.

[39] Yu Y., Liu Q., Wang C., Zhang D., Jiang B., Shan Y., Fu F., Ding S. (2022). Zein/Pullulan Complex Colloidal Particle-Stabilized Pickering Emulsions for Oral Delivery of Polymethoxylated Flavones: Protection Effect and In Vitro Digestion. J. Sci. Food Agric..

[40] Meng R., Wu Z., Xie Q.-T., Zhang B., Li X.-L., Liu W.-J., Tao H., Li P.-J. (2020). Zein/Carboxymethyl Dextrin Nanoparticles Stabilized Pickering Emulsions as Delivery Vehicles: Effect of Interfacial Composition on Lipid Oxidation and In Vitro Digestion. Food Hydrocoll..

[41] Ji Y., Han C., Liu E., Li X., Meng X., Liu B. (2022). Pickering Emulsions Stabilized by Pea Protein Isolate-Chitosan Nanoparticles: Fabrication, Characterization and Delivery EPA for Digestion In Vitro and In Vivo. Food Chem..

[42] Zhou S., Han L., Lu K., Qi B., Du X., Liu G., Tang Y., Zhang S., Li Y. (2022). Whey Protein Isolate–Phytosterols Nanoparticles: Preparation, Characterization, and Stabilized Food-Grade Pickering Emulsions. Food Chem..

[43] Huang M., Wang Y., Ahmad M., Ying R., Wang Y., Tan C. (2021). Fabrication of Pickering High Internal Phase Emulsions Stabilized by Pecan Protein/Xanthan Gum for Enhanced Stability and Bioaccessibility of Quercetin. Food Chem..

[44] Zhou F.Z., Zeng T., Yin S.W., Tang C.H., Yuan D.B., Yang X.Q. (2018). Development of Antioxidant Gliadin Particle Stabilized Pickering High Internal Phase Emulsions (HIPEs) as Oral Delivery Systems and the In Vitro Digestion Fate. Food Funct..

[45] Yi J., Gan C., Wen Z., Fan Y., Wu X. (2021). Development of Pea Protein and High Methoxyl Pectin Colloidal Particles Stabilized High Internal Phase Pickering Emulsions for β-Carotene Protection and Delivery. Food Hydrocoll..

